# Vitamin D levels in Indian systemic lupus erythematosus patients: association with disease activity index and interferon alpha

**DOI:** 10.1186/ar4479

**Published:** 2014-02-10

**Authors:** Manamita Mandal, Rina Tripathy, Aditya K Panda, Sarit S Pattanaik, Simanchal Dakua, Anjan Kumar Pradhan, Soumen Chakraborty, Balachandran Ravindran, Bidyut K Das

**Affiliations:** 1 Department of Medicine, SCB Medical College, Medical College Road, Cuttack, OD 753007, India; 2 Department of Biochemistry, SCB Medical College, Medical College Road, Cuttack, OD 753007, India; 3 Infectious Disease Biology Group, Institute of Life Sciences, Nalco Nagar Road, Bhubaneswar, OD 751023, India; 4 Department of Gene Function and Regulation, Institute of Life Sciences, Nalco Nagar Road, Bhubaneswar, OD 751023, India

## Abstract

**Introduction:**

Low levels of vitamin D have been associated with several autoimmune disorders including multiple sclerosis, rheumatoid arthritis, type 1 diabetes and systemic lupus erythematosus (SLE). The major source of vitamin D is sunlight but exposure of SLE patients to UV rays has been shown to exacerbate disease pathology. Studies in various populations have shown an association between low vitamin D levels and higher SLE disease activity.

**Methods:**

We enrolled 129 patients who fulfilled American College of Rheumatology criteria in the study. There were 79 treatment-naïve cases and 50 patients who were under treatment for underlying SLE. There were 100 healthy subjects from similar geographical areas included as controls. Plasma 25-OH vitamin D_3_ and interferon (IFN)-α levels were quantified by enzyme-linked immunosorbent assay (ELISA). The gene expression level of IFN-α was determined by quantitative real-time reverse transcriptase polymerase chain reaction (RT-PCR).

**Results:**

Plasma 25-OH vitamin D_3_ significantly correlated in an inverse manner with systemic lupus erythematosus disease activity index (SLEDAI) scores (*P* <0.0001, r = -0.42), anti-dsDNA (*P* <0.0001, r = -0.39), plasma IFN-α (*P* <0.0001, r = -0.43) and levels of IFN-α gene expression (*P* = 0.0009, r = -0.45). Further, plasma levels of IFN-α positively correlated with gene expression of IFN-α (*P* <0.0001, r = 0.84). Treatment-naïve SLE patients displayed significantly higher plasma levels of IFN-α compared to patients under treatment (*P* <0.001) and controls (*P* <0.001).

**Conclusions:**

These results suggest an important role of vitamin D in regulating disease activity in SLE patients and the need to supplement vitamin D in their treatment.

## Introduction

Systemic lupus erythematosus (SLE) is an autoimmune disorder which appears in a group of individuals and which is related to several factors, including environmental and host genetics that contribute to the development of the disease [1]. Patients with SLE develop an immune response against numerous, mostly intracellular self-antigens. This results in formation of immune complexes that get deposited in vascular beds in most organs of the body. Immune complex deposition causes local inflammation and tissue damage that probably amplify the autoimmune response [2]. This has serious consequences on the outcome of the disease.

The importance of vitamin D in various autoimmune disorders has been reported. Vitamin D deficiency has been associated with multiple sclerosis (MS), rheumatoid arthritis (RA), type 1 diabetes mellitus, inflammatory bowel disease (IBD), mixed connective tissue disease, autoimmune thyroid disease, scleroderma and SLE [3-5]. Vitamin D supplementation improves disease outcome in various animal models of MS [6], RA [7], type 1 diabetes mellitus [8], IBD [9], autoimmune encephalomyelitis [10] and SLE [11]. The role of vitamin D in murine models of SLE has been investigated to a limited degree. Administration of vitamin D and its synthetic analogs to murine models has resulted in improved dermatological manifestations [11], reduced proteinuria [12] and increased survival [12,13]. An earlier report highlighted vitamin D_3_ insufficiency in two-thirds, and deficiency (<10 ng/ml) in approximately one-fifth of SLE patients [14]. In addition, serum vitamin D_3_ (25-OH) levels have been found to correlate inversely with SLE disease activity index (SLEDAI) scores [15-17].

The major source of vitamin D is the conversion of 7-dehydrocholesterol to previtamin D_3_ in the skin when exposed to solar ultraviolet radiation [18]. Previtamin D_3_ then gets converted to vitamin D_3_ (cholecalciferol) through a heat-mediated process in the skin [18]. A lesser amount of vitamin D_3_ (25-OH) is obtained from foods that supply less than 20% of the body’s requirements. Vitamin D_3_ undergoes two hydroxylations to achieve its functional form. The first hydroxylation occurs in the liver resulting in 25-hydroxyvitamin D (25(OH)D_3_) or calcidiol, which is normally quantified for evaluating vitamin D status, and the second hydroxylation takes place in the kidney to its active form 1,25-dihydroxyvitamin D3 (1, 25(OH)_2_D) [18]. In addition to the liver and kidney, hydroxylation of vitamin D_3_ also occurs in the lymph nodes and skin [19].

Several studies worldwide have investigated the role of vitamin D_3_ in the pathogenesis of SLE. However, to date, there have been no reports from an Indian population. Although the prevalence of SLE in India is rare (3 per 100,000) [20], the survival rates of these patients (5-year: 70%; 10-year: 50%) are low compared to Western cohorts [21,22]. Interestingly, vitamin D_3_ insufficiency or deficiency appears to be widespread in the Indian subcontinent [23], which makes it important to analyze its role in the background of SLE from an Indian cohort. We have addressed this issue in a tertiary-care, hospital-based, case-control study, to assess the role of vitamin D_3_ in SLE in a cohort from eastern India.

## Methods

### Subjects

The patients recruited for the study were all inpatients, admitted to the Department of Medicine, under the Clinical Immunology and Rheumatology unit of SCB Medical College, Cuttack, Odisha. As described earlier [24-26], diagnosis of SLE was based on the revised American College of Rheumatology (ACR) classification criteria [27]. After a detailed clinical examination and laboratory investigation, the clinical manifestations were categorized. The clinical profiles of 129 SLE patients are summarized in Table 1. Since, SLE affects women primarily [28], 50 age-matched healthy females (medical students: HCA) and 50 healthy subjects from similar geographical areas (HCB) were included as healthy controls (HC). None of the controls reported any history of autoimmune disorder. About 5 ml blood in EDTA was collected from each participant. The study was approved by the institutional ethics committee of SCB Medical College, Cuttack. Informed consent was obtained from each patient and healthy control.

**Table 1:** Clinical characteristics of SLE patients and healthy controls

**Clinical profiles**	**SLE (n = 129)**	**Healthy controls (n = 100)**	
**Sex (male/female)**	4/125	26/74	
**Age in years (mean ± SD)**	28.14 ± 8.43	31.18 ± 5.32	
**Duration of disease years (mean ± SD)**	2.90 ± 2.66	-	
**SLEDAI scores (mean ± SD)**	18.36 ± 6.73	-	
**Photosensitivity rash**	34 (26)	-	
**Malar rash**	73 (57)	-	
**Discoid rash**	14 (11)	-	
**Oral ulcer**	76 (59)	-	
**Arthritis**	77 (60)	-	
**NPSLE**	11 (9)	-	
**Myocarditis**	3 (2)	-	
**Serositis**	7 (5)	-	
**Nephritis**	46 (37)	-	
**Vasculitis**	17 (13)	-	
**Treatment details of patients under therapy at the time of recruitment to the study (n = 50)**			
	Medicine	Quantity	Number of patient treated (%)
	Prednisolone; mean (range)	18.99 mg (5-50 mg)	50 (100)
	Hydroxychloroquine	6.5 mg/kg body weight	50 (100)
	Calcium	1 g/day	50 (100)
	Vitamin D_3_	250-500 IU/day	50 (100)
	Azathioprine	50-100 mg/day	4 (8)
	Mycophenolate mofetil	2 gm/day	4 (8)

Note: data are number (%) of participants unless otherwise specified. SLE, systemic lupus erythematosus; SLEDAI, SLE disease activity index; NPSLE*,* neuropsychiatric systemic lupus erythematosus.

### 25-OH vitamin D quantification in plasma

The plasma levels of 25-OH Vitamin D were quantified by enzyme-linked immunosorbent assay (ELISA) kit (CPC, Euroimmun, Lübeck, Germany) according to the manufacturer’s instructions. Vitamin D deficiency was defined as plasma levels of 25-OH vitamin D <10 ng/ml and insufficiency as 10 to 30 ng/ml [18].

### Quantification of plasma interferon alpha

Plasma levels of interferon (IFN)-α were measured by ELISA kit (Bender MedSystems Inc., Burlingame, CA, USA) according to the manufacturer’s protocol.

### RNA extraction and reverse transcription

According to the manufacturer’s instructions, total RNA was isolated from 250 μl of whole blood by TRIzol LS reagent (Invitrogen, Carlsbad, CA, USA). RNA concentration was determined by spectrophotometry using an Implen NanoPhotometer (Implen, Munich, Germany). To remove any traces of genomic DNA, 1 μg of total RNA was then treated with 2U DNase (Sigma-Aldrich, St Louis, MO, USA) for 30 min at 37°C. DNase-treated RNA was reverse transcribed with a hexamer primer using a First Strand cDNA Synthesis kit (Thermo Fisher Scientific, Waltham, MA, USA), according to the manufacturer’s instructions. Once the cDNA was synthesized, its fidelity was tested by PCR and stored at -70°C.

### Real-time PCR assay

Real-time PCR assay of IFN-α was carried out as described earlier [29]. Briefly, reactions were set up in a total volume of 20 μl using 2 μl of cDNA, 10 μl of MESA GREEN qPCR MasterMix Plus (Eurogentec, Seraing, Belgium) and 10 picomole each of gene-specific primer (IFN-α (sense: 5′-TTCCTCCTGYYTGAWGGACAGA-3; antisense: 5′-GATCTCATGATTTCTGCTCTGACA-3′), glyceraldehyde-3 phosphate dehydrogenase (G3PDH) was taken as control (sense: 5′-GGTATCGTGGAAGGACTCATGAC-3′; antisense: 5′-ATGCCAGTGAGCTTCCCGTTCAGC-3′)) and performed in the MJ Research DNA Engine Opticon Real-Time Thermal Cycler (MJ Research, Waltham, MA, USA).The cycling conditions were: 95°C for 4 min; 35 cycles of 95°C for 30 s, 55°C for 30 s and 72°C for 30 s with a single fluorescence measurement; a final elongation step was carried out at 72°C for 10 min. Specificity of the PCR products was confirmed by analysis of the dissociation curve. The melting curve program consisted of temperatures between 55 and 95°C with a heating rate of 0.1°C/s and a continuous fluorescence measurement. Additionally, the amplicons’ expected size and the absence of nonspecific products were confirmed by analysis of the real-time PCR products in 1% agarose gel in 1 × TBE, stained with ethidium bromide and visualized under ultraviolet light (expected product size of IFN-α: 375 bp and G3PDH: 187 bp). IFN-α gene expression in each sample was calculated by the 2^-ΔCt^ method (ΔCt = Ct of IFN-α – Ct of GAPDH) [30].

### Statistical analysis

All statistical analysis was performed by GraphPad prism 5.01 (GraphPad Software, San Diego, CA, USA). Distribution of plasma 25-OH vitamin D_3_ and IFN-α in treatment-naïve SLE patients, controls and treated patients were assessed by D’Agostino-Pearson omnibus normality test. Based on the results of the normality test, the association of 25-OH vitamin D_3_ and IFN-α with clinical disease was analyzed by analysis of variance (ANOVA) or Kruskal-Wallis test followed by an appropriate post test. Correlation of 25-OH vitamin D_3_ with double-stranded (ds)DNA, SLEDAI scores and IFN-α was analyzed by Spearman’s correlation test. Further correlation of IFN-α gene expression with plasma IFN-α and 25-OH vitamin D_3_ levels was analyzed by Spearman’s correlation test. A *P* value <0.05 was considered as significant.

## Results

### Clinical characteristics of SLE patients

One hundred and twenty-nine patients were enrolled in the current study. Baseline characteristics are shown in Table 1. There were 125 (97%) females and 4 (3%) males with a mean age (standard deviation) of 28.14 (8.43) years. The mean duration of disease (standard deviation) was 2.90 years (2.66). Out of the 129 SLE patients, 50 patients included in the study were already on treatment for SLE and were also receiving supplements of oral calcium and vitamin D_3_ at the time of blood collection (Table 1). The other 79 patients were treatment-naive cases, undiagnosed earlier and the details of the treatment received for their complaints before hospitalization were not known since the patients had not maintained any records. The clinical profiles of patients were as follows: photosensitivity rash (26%), malar rash (57%), discoid rash (11%), oral ulcer (59%), arthritis (60%), neuropsychiatric disease (9%), myocarditis (2%), serositis (5%), nephritis (37%) and vasculitis (13%) (Table 1).

### Plasma 25-OH vitamin D_3_ levels in SLE patients and healthy controls

**Figure 1:**
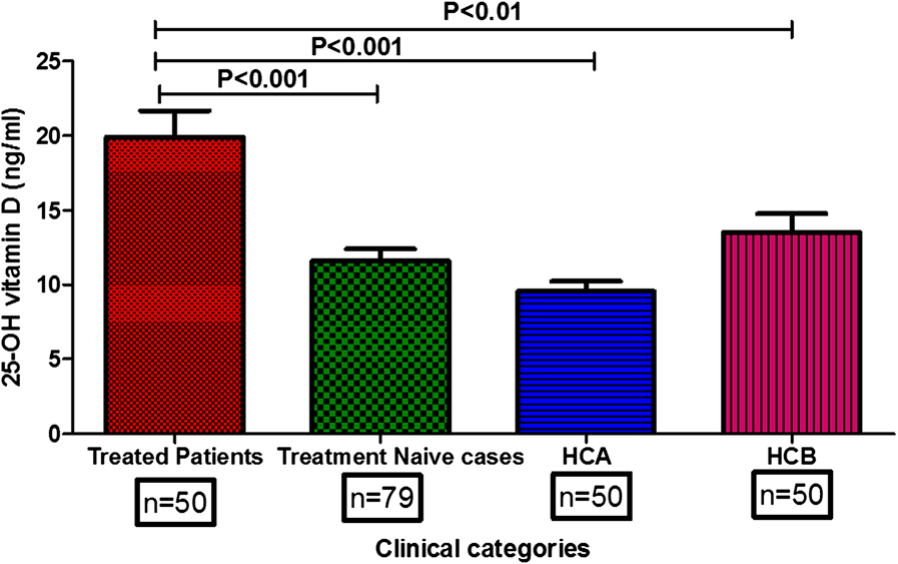
**25-OH vitamin D**_**3 **_**levels in SLE patients and healthy controls.** Plasma samples from SLE patients (treatment-naïve case (n = 79); treated patients (n = 50)) and healthy controls (HCA: medical students (n = 50); HCB: controls from same locality (n = 50)) were quantified by ELISA according to the manufacturer’s instructions. Treated SLE patients displayed significantly higher concentrations of plasma 25-OH vitamin D_3_ levels compared to treatment-naïve patients (*P* <0.001) and controls (HCA: (*P* <0.001); HCB: (*P* <0.01)). SLE, systemic lupus erythematosus.

Plasma levels of 25-OH vitamin D_3_ in SLE patients and healthy controls were quantified by ELISA and the results are shown in Figure 1. Patients under vitamin D_3_ supplementation (treated cases) displayed significantly higher levels of 25-OH vitamin D_3_ compared to treatment-naïve patients (*P* <0.001) and healthy controls (*P* <0.001). The levels of plasma 25-OH vitamin D_3_ in treatment-naïve SLE patients and healthy controls were comparable.

### Vitamin D_3_ levels negatively correlated with SLEDAI scores and anti-dsDNA

**Figure 2:**
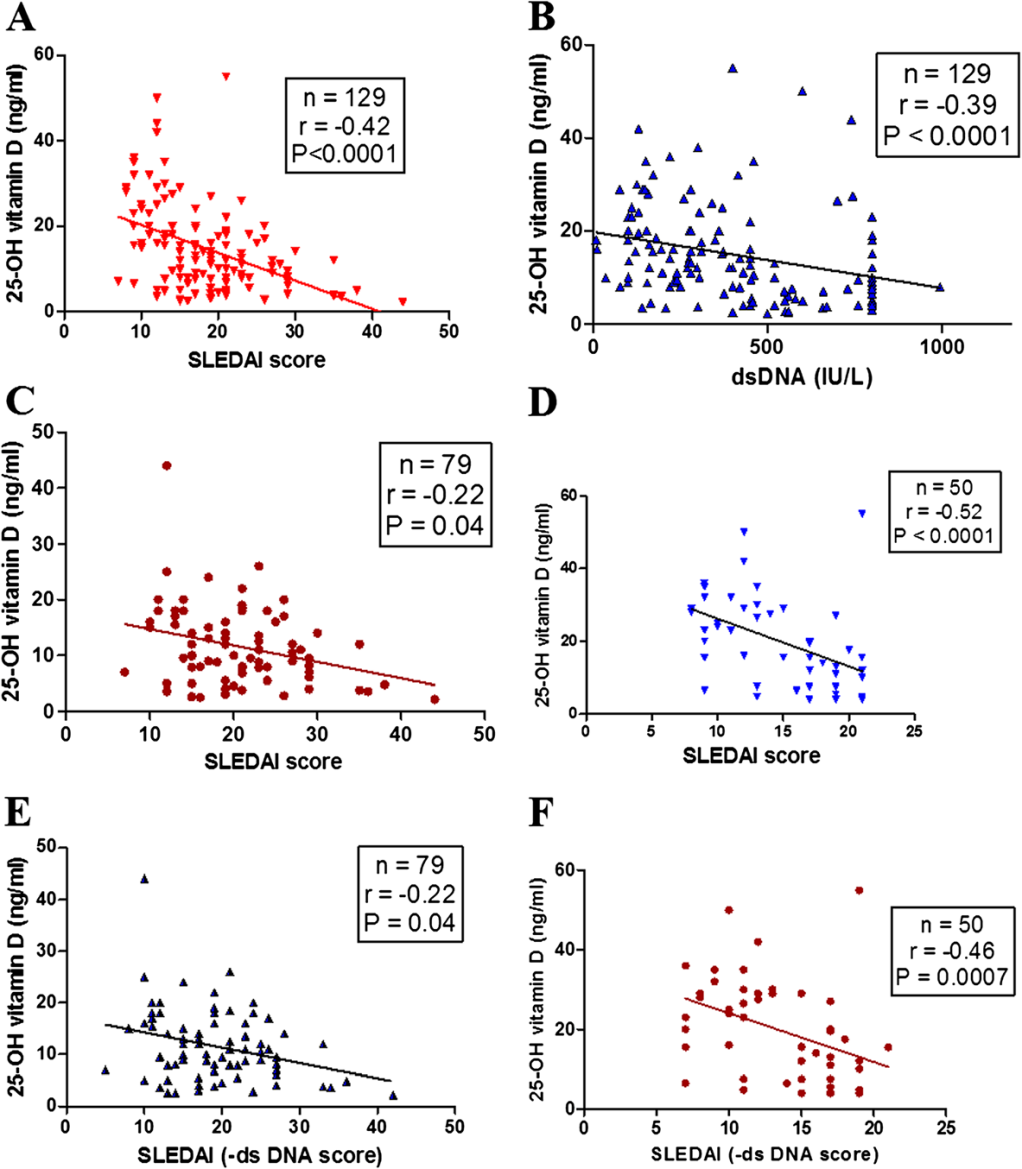
**Correlation of 25-OH vitamin D**_**3 **_**with SLEDAI scores and anti-dsDNA levels.** Plasma 25-OH vitamin D_3_ levels of SLE patients correlated negatively with SLEDAI scores **(A)** and anti-dsDNA **(B)**. SLE patients were categorized into two groups: treatment-naïve cases and patients under treatment. In both groups plasma levels of 25-OH vitamin D_3_ negatively correlated with SLEDAI scores **(C** and **D)**. Modified SLEDAI scores (eliminating the anti-dsDNA score of 2) and its correlation with plasma levels of 25-OH vitamin D_3_ were analyzed. Similar to earlier observations, SLEDAI scores (-anti-dsDNA) negatively correlated with 25-OH vitamin D_3_ levels in both treatment-naïve cases **(E)** and treated patients **(F)**. Dots represent individual samples. Correlation analysis was performed by Spearman’s correlation coefficient. A *P* value less than 0.05 was considered as significant. SLEDAI, systemic lupus erythematosus disease activity index; SLE, systemic lupus erythematosus; dsDNA, double-stranded DNA.

Analysis of data in SLE patients revealed a significant negative correlation between plasma 25-OH vitamin D_3_ levels with SLEDAI scores (*P* <0.0001, r = -0.42) (Figure 2A) and anti-dsDNA (*P* <0.0001, r = -0.39) (Figure 2B). Further, SLE patients were categorized into two groups: treatment-naïve and those under treatment. As shown in Figure 2C and D, irrespective of treatment status, the plasma levels of 25-OH vitamin D_3_ negatively correlated with SLEDAI scores. In addition, a further analysis of the relationship between 25-OH vitamin D_3_ and modified SLEDAI scores (eliminating the anti-dsDNA positive score of 2 from SLEDAI), revealed identical results in both the groups (Figures 2E and F). These findings indicate a significant association between 25-OH vitamin D_3_ and disease activity in SLE.

### Correlation between 25-OH vitamin D_3_ and IFN-α

**Figure 3:**
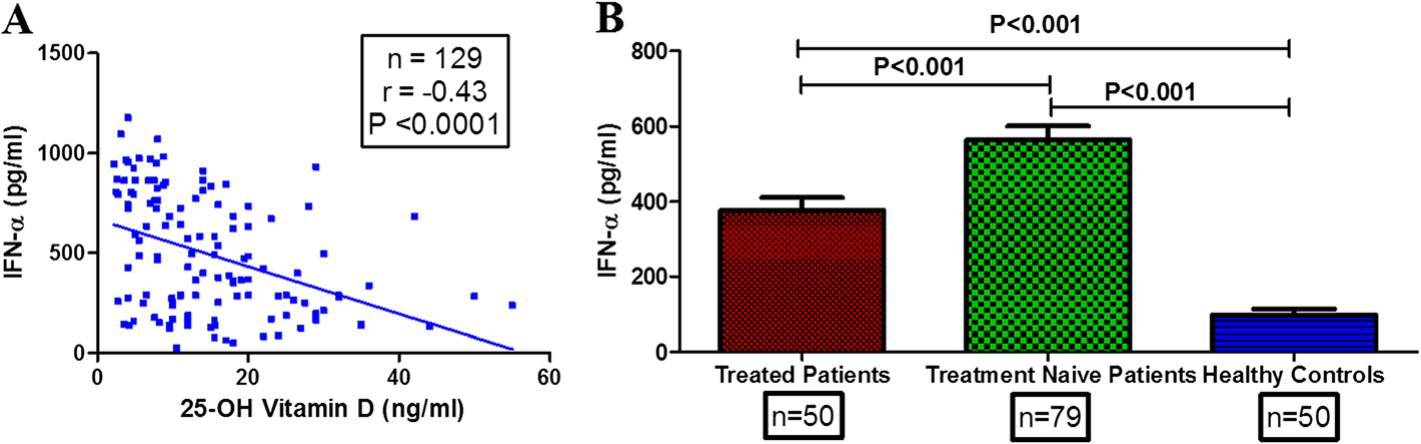
**Correlation of plasma IFN-α with 25-OH vitamin D**_**3 **_**and its levels in SLE patients and controls. (A)** Plasma 25-OH vitamin D_3_ levels correlated negatively with IFN-α levels. Dots represent individual samples. Correlation analysis was performed by Spearman’s correlation coefficient. **(B)** Treatment-naïve patients displayed significantly higher levels of IFN-α compared to treated cases and healthy controls. Mean plasma levels of IFN-α in different clinical categories were compared by ANOVA followed by Tukey’s multiple comparisons test. A *P* value less than 0.05 was considered as significant. IFN-α, interferon alpha; SLE, systemic lupus erythematosus.

The role of IFN-α in SLE has been clearly documented and its significant correlation with SLEDAI scores has been demonstrated in patients from different populations [31,32]. As shown in Figure 3A, a significant negative correlation was observed between 25-OH vitamin D_3_ levels and plasma IFN-α (*P* <0.0001, r = -0.43). Treatment-naïve patients displayed higher levels of plasma IFN-α compared to SLE patients on treatment (*P* <0.001) and healthy controls (*P* <0.001) (Figure 3B) suggesting an important role of IFN-α in modulating disease activity.

### Correlation of IFN-α gene expression with plasma IFN-α and 25-OH vitamin D_3_ levels

**Figure 4:**
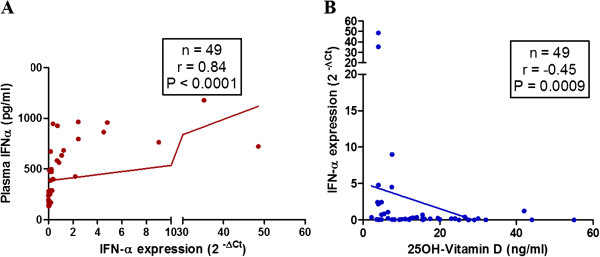
**Correlation of IFN-α gene expression with plasma IFN-α levels and 25-OH vitamin D**_**3**_**.** Plasma levels of IFN-α and gene expression levels were quantified by ELISA and RT-PCR respectively. **(A)** Plasma levels of IFN-α positively correlated with gene expression (2 ^– Δct^) of IFN-α. **(B)** IFN-α gene expression (2 ^– Δct^) correlated negatively with plasma levels of 25-OH vitamin D_3_. Dots represent individual samples. Correlation analysis was performed by Spearman’s correlation coefficient. A *P* value less than 0.05 was considered as significant. IFN-α, interferon alpha.

To validate the robustness of IFN-α data, considering that it is an evanescent cytokine, we quantified IFN-α gene expression by RT-PCR in SLE patients (n = 49) and correlated the values with plasma levels of IFN-α and 25-OH vitamin D_3_. As shown in Figure 4A, a strong positive correlation was observed between IFN-α plasma levels and its gene expression (*P* <0.0001, r = 84). In addition, IFN-α gene expression negatively correlated with plasma 25-OH vitamin D_3_ (*P* = 0.0009, r = -45) (Figure 4B).

### Association of plasma IFN-α with SLE disease severity

**Figure 5:**
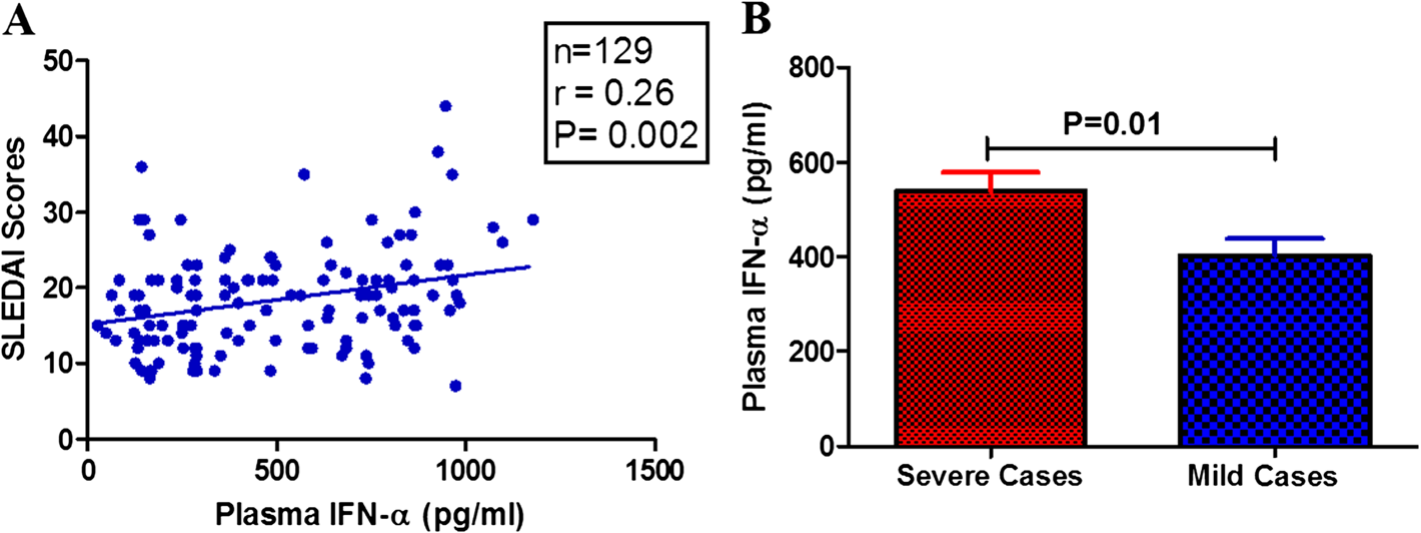
**Correlation of plasma IFN-α with SLEDAI scores and association with organ involvement. (A)** Plasma levels of IFN-α positively correlated with SLEDAI scores. Dots represent individual samples. Correlation analysis was performed by Spearman’s correlation coefficient. A *P* value less than 0.05 was considered as significant. **(B)** Based on clinical phenotype SLE patients were categorized into two broad groups and plasma levels of IFN-α were compared. Patients with major disease manifestation displayed significantly higher plasma levels of IFN-α than those with minor disease manifestation. Mean plasma levels of IFN-α were compared by unpaired *t* test and a *P* value less than 0.05 was taken as significant. IFN-α, interferon alpha; SLEDAI, systemic lupus erythematosus disease activity index; SLE, systemic lupus erythematosus.

Role of IFN-α in the pathogenesis of SLE is an important issue that is being investigated [32]. We analyzed the association of IFN-α with disease severity. As shown in Figure 5A, plasma levels of IFN-α positively correlated with SLEDAI scores (r = 0.26, *P* = 0.002) and patients with severe phenotype displayed significantly higher levels of IFN-α compared to those with mild disease manifestations (*P* = 0.01) (Figure 5B). However, duration of disease did not correlate with plasma IFN-α levels (data not shown). We also observed a significant inverse correlation between plasma 25-OH vitamin D_3_ and IFN-α levels. This correlation held true while analyzing the association between 25-OH vitamin D_3_ levels and IFN-α gene expression.

## Discussion

The role of vitamin D_3_ in autoimmune disorders has been the subject of several studies with regard to its importance as an immune regulator [33]. This is the first study from India to demonstrate an association between vitamin D_3_ and SLE, highlighting its significant inverse correlation with SLEDAI scores, anti-dsDNA and IFN-α. These are markers of disease activity and IFN-α is closely associated with disease pathogenesis.

Low levels of vitamin D_3_ in SLE patients have been reported compared to healthy controls in different populations [34]. Interestingly, mean plasma levels of 25-OH vitamin D3 were not significantly different among treatment-naïve SLE cases (11.61 ng/ml), healthy medical students (9.55 ng/ml) and other healthy controls from same locality (13.36 ng/ml). Vitamin D_3_ insufficiency has been reported to be widely prevalent in the Indian subcontinent irrespective of the social class [23]. Two groups of healthy controls were analyzed, which included medical students (HCA), who led a lifestyle marked by poor exposure to sunlight and irregular dietary habits, and a group of healthy subjects from the same locality (HCB). Interestingly, 63% of healthy medical students were deficient and 37% were insufficient of vitamin D_3_. Furthermore, 94% of the other groups of healthy controls were either deficient or insufficient of vitamin D_3_. This was an important observation considering India being a tropical country with lots of sunshine. However, the facts were contrary and several hypotheses have been discussed to explain the discrepancy. Higher melanin concentration in the skin [35], current lifestyle changes, avoidance of sunlight and poor food habits are some of the causes attributed to the widespread prevalence of low vitamin D_3_ among Indians. Low vitamin D_3_ may not be cause for development of SLE but persons with low serum levels are likely to suffer from severe disease. The current cross-sectional study does not address the issue of cause and effect relationship between vitamin D_3_ and SLE.

There are several interesting observations in the current study that points to an important role for vitamin D_3_ in disease modulation. One of them being a significant inverse correlation between vitamin D_3_ and SLEDAI scores and the other association is between vitamin D_3_ and anti-dsDNA. Association between plasma vitamin D_3_ and SLEDAI scores has not been uniform across observations: several studies have reported a negative correlation [15-17], while others have found none [36-39].

One of the important functions of vitamin D_3_ is maintenance of homeostasis of B cells [40]. Low levels of vitamin D_3_ contribute to hyperactivity of B cells and enhanced production of autoantibodies [41]. Furthermore, vitamin D_3_ is known to modulate various immunological pathways [33] and thus could have a defining role in the development, progression and pathogenesis of SLE. Vitamin D_3_ also inhibits differentiation of dendritic cells (DCs) and T-helper cells (CD4+) [42], enhances T regulatory cell proliferation and suppresses release of inflammatory mediators [43], which collectively help in control of autoimmune disorders.

In recent years, the role of interferon in the pathogenesis of lupus has been widely investigated. Higher levels of IFN-α were observed in our SLE patients compared to healthy controls, corroborating earlier observations [32,44-46]. The interferon levels were significantly low in patients under treatment compared to treatment-naïve cases, supporting its possible role in disease modulation. Furthermore, IFN-α could be a marker of disease activity and low levels in treated patients could indicate response to therapy.

Interestingly, our study revealed a strong negative correlation of vitamin D_3_ with IFN-α (*P* <0.0001, r = -0.52). The robustness of the assay was validated by assessment of IFN-α gene expression, which corroborated with the earlier observations on the association between plasma IFN-α and vitamin D_3_. There are no reports in the literature assessing the association between IFN-α and vitamin D_3_.

In active SLE overexpression of interferon-inducible genes (IFN signature) has been reported [47]. The major source of IFN-α in SLE patients are activated DCs. Maturation/activation of DCs and production of IFN-α has been observed to be inhibited by vitamin D in *in vitro* studies [48,49]. A direct role for vitamin D_3_ in modulating lupus activity has been demonstrated in animal models [11-13]. Our observations, although cross-sectional, and studies on experimental models, provide evidence for a disease-modulating role for vitamin D_3_, which could be a promising therapeutic adjunct in the treatment of SLE. In view of the limited number of drugs available for the treatment of lupus and the low cost of vitamin D_3_ therapy, there is a strong case for its use routinely.

## Conclusions

To conclude, vitamin D deficiency is prevalent among healthy Indians as well as among SLE patients. The significant inverse correlation of vitamin D_3_ with SLEDAI scores, anti-dsDNA and IFN-α highlights its immune-modulatory role contributing to disease outcome. Although the present study indicates a necessity for vitamin D_3_ supplementation in the management of SLE patients, larger randomized controlled trials would be necessary to define the daily requirement and optimum blood levels of vitamin D_3_ that are effective in influencing disease outcome.

## Abbreviations

ACR: American College of Rheumatology; C3: complement component 3; C4: complement component 4; DCs: dendritic cells; dsDNA: double-stranded DNA; ELISA: enzyme-linked immunosorbent assay; HC: healthy controls; IBD: inflammatory bowel disease; IFN-α: interferon alpha; MS: multiple sclerosis; NPSLE: neuropsychiatric systemic lupus erythematosus; RT-PCR: real-time polymerase chain reaction; RA: rheumatoid arthritis; SLE: systemic lupus erythematosus; SLEDAI: systemic lupus erythematosus disease activity index.

## Competing interests

The authors declare that they have no competing interests.

## Authors’ contributions

MM, SD and SSP were involved in samples collection, data management and clinical categorization of samples. AdKP carried out quantification of IFN-α by ELISA, real-time PCR, data interpretation and wrote the first draft of the manuscript. AnKP and SC quantified IFN-α gene expression and interpreted the results. RT performed all routine tests including measurement of vitamin D. RT, BR and BKD made a contribution in the design, data interpretation, work supervision and critically revising the manuscript. All authors read and approved the manuscript.
